# OsMYB103L, an R2R3-MYB transcription factor, influences leaf rolling and mechanical strength in rice (*Oryza sativa* L.)

**DOI:** 10.1186/1471-2229-14-158

**Published:** 2014-06-06

**Authors:** Chunhua Yang, Dayong Li, Xue Liu, Chengjun Ji, Lili Hao, Xianfeng Zhao, Xiaobing Li, Caiyan Chen, Zhukuan Cheng, Lihuang Zhu

**Affiliations:** 1 State Key Laboratory of Plant Genomics and National Center for Plant Gene Research, Institute of Genetics and Developmental Biology, Chinese Academy of Sciences, Beijing 100101, China; 2 CAS Key Laboratory of Genome Sciences and Information, Beijing Institute of Genomics, Chinese Academy of Sciences, Beijing 100101, China; 3 Department of Ecology, Peking University, Beijing 100871, China; 4 Key Laboratory of Agro-Ecological Processes in Subtropical Region, Institute of Subtropical Agriculture, Chinese Academy of Sciences, Changsha 410125, China

**Keywords:** *OsMYB103L*, Leaf rolling, MYB transcription factor, Cellulose

## Abstract

**Background:**

The shape of grass leaves possesses great value in both agronomy and developmental biology research. Leaf rolling is one of the important traits in rice (*Oryza sativa* L*.*) breeding. MYB transcription factors are one of the largest gene families and have important roles in plant development, metabolism and stress responses. However, little is known about their functions in rice.

**Results:**

In this study, we report the functional characterization of a rice gene, *OsMYB103L*, which encodes an R2R3-MYB transcription factor. OsMYB103L was localized in the nucleus with transactivation activity. Overexpression of *OsMYB103L* in rice resulted in a rolled leaf phenotype. Further analyses showed that expression levels of several cellulose synthase genes (*CESAs*) were significantly increased, as was the cellulose content in *OsMYB103L* overexpressing lines. Knockdown of *OsMYB103L* by RNA interference led to a decreased level of cellulose content and reduced mechanical strength in leaves. Meanwhile, the expression levels of several *CESA* genes were decreased in these knockdown lines.

**Conclusions:**

These findings suggest that *OsMYB103L* may target *CESA* genes for regulation of cellulose synthesis and could potentially be engineered for desirable leaf shape and mechanical strength in rice.

## Background

Leaves are the main organs of photosynthesis and play a crucial role in plant development. Leaf rolling is a frequently observed phenotype in fields and rice mutant populations [1-3]. The appropriate leaf rolling has been regarded as one of the most important elements in rice ideotype breeding as it improves photosynthetic efficiency, accelerates dry-matter accumulation, reduces solar radiation on leaves, decreases leaf transpiration under drought stress and raises grain yield [2,4-9]. However, severe leaf rolling could lead to growth retardation, developmental defects and crop yield reduction.

To date, many studies have been performed to characterize the genes controlling leaf rolling by analyses of the rolled leaf mutants in rice. Twelve rolled leaf (*rl*) mutants in rice are reported and the corresponding genes are mapped genetically, of which, six *rl* genes are mapped on rice chromosomes by morphological markers and the rest are directly mapped in rice genome by molecular markers [10-14]. Among these 12 *rl* mutants, *rl9* is the first to be cloned and analyzed in detail. *ROLLED LEAF9* (*RL9*)/*SHALLOT-LIKE1* (*SLL1*) encodes a KANADI transcription factor, and *rl9/sll1* mutants display extremely incurved leaves due to the defective development of sclerenchymatous cells on the abaxial side [1,15]. Some other genes are also found to be related to leaf rolling in rice. Loss-of-function of *OsCSLD4* gene, which encodes a cellulose synthase-like protein, results in phenotypes of reduced leaf width and semi-rolled leaves, possibly due to the significantly smaller bulliform cells in mutants [16-19]. Loss-of-function of *CONSTITUTIVELY WILTED1* (*OsCOW1*)/*NARROW LEAF7* (*NAL7*), encoding a member of the YUCCA protein, causes rice leaves to roll upward [20,21]. Loss-of-function of *CURLY FLAG LEAF* (*CFL1*), encoding a WW domain protein, induces rolled leaves which have a wrinkled epidermis and contain papillae with reduced wax content [22]. *ROLLED LEAF 14* (*RL14*), encoding a 2OG-Fe (II) oxygenase family protein, is reported to modulate rice leaf rolling by affecting secondary cell wall formation and bulliform cell development [2]. Mutation of *SEMI-ROLLED LEAF 1* (*SRL1*), encoding a putative GPI-anchored protein, results in leaves rolled adaxially in rice due to the increased number of bulliform cells at the adaxial cell layers [23]. In addition, recent studies show that overexpression of rice gene *OsAGO7,* which encodes an Argonaute (AGO) family member, results in the leaf blades curling upward [3]. *OsHB1* is a member of the Class III homeodomain leucine zipper family of genes, overexpression of its *microRNA166*-resistant version could result in adaxially rolled leaves due to the reduced sclerenchyma and formation of bulliform cells on the abaxial side [24]. Taken together, the major factors possibly affecting leaf rolling include the alteration of osmotic pressure and/or turgidity in bulliform cells, the impairment of cell polarity establishment and cell differentiation. However, the mechanisms underlying leaf rolling in rice and, particularly, the involved transcription factors remain largely unknown [1].

MYB proteins belong to a super-family of transcription factors, which prevail in all eukaryotes and share a highly conserved DNA-binding domain, MYB [25-27]. Depending on the number of adjacent repeats in the MYB domain, MYB proteins can be classified into four subfamilies, including 1R-MYB, R2R3-MYB, 3R-MYB, and 4R-MYB [26,28]. Among them, R2R3-MYB subfamily is a typical representative one, whose members contain two MYB repeats in their DNA-binding domains [29,30]. MYB proteins in plants are involved in a variety of plant-specific processes, including primary and secondary metabolism, cell fate and identity, developmental processes, and responses to various stresses [25-27,31-40]. Genome-wide analysis shows that there are approximately 183 MYB genes in rice, of which 109 encode R2R3-MYB proteins [41,42]. So far, only a few number of MYB proteins, including OsGAMYB, OsMYB2, OsMyb4, OsMYB3R-2, CSA*,* MYBS3 and OsMYB2P-1 [43-49], have been functionally characterized. The functions of most of MYB proteins are still unknown in rice.

To characterize functions of MYB transcription factors in rice, we overexpressed several MYB genes in Kasalath, an *indica* cultivar, with the transgene constructs containing the full-length cDNAs of rice MYB genes, driven by maize *ubiquitin* promoter. Of the transgenic lines, one line overexpressing the full-length cDNA of *Os08g05520*, designated as *OsMYB103L* hereafter, displays a rolled leaf phenotype. *OsMYB103L* encodes an R2R3-MYB transcription factor. Our study shows that it localizes in the nucleus and possesses transcriptional activity. We detail the phenotypes of *OsMYB103L* overexpressing (OE) and RNA interference (RNAi) knockdown plants, including the altered leaf shape, the changed cellulose content, and the impaired mechanical strength. The roles of *OsMYB103L* in leaf shape formation and cellulose synthesis are discussed. We propose the potential application of *OsMYB103L* in molecular breeding of rice.

## Results

### *OsMYB103L* encodes an R2R3-MYB transcription factor

**Figure 1:**
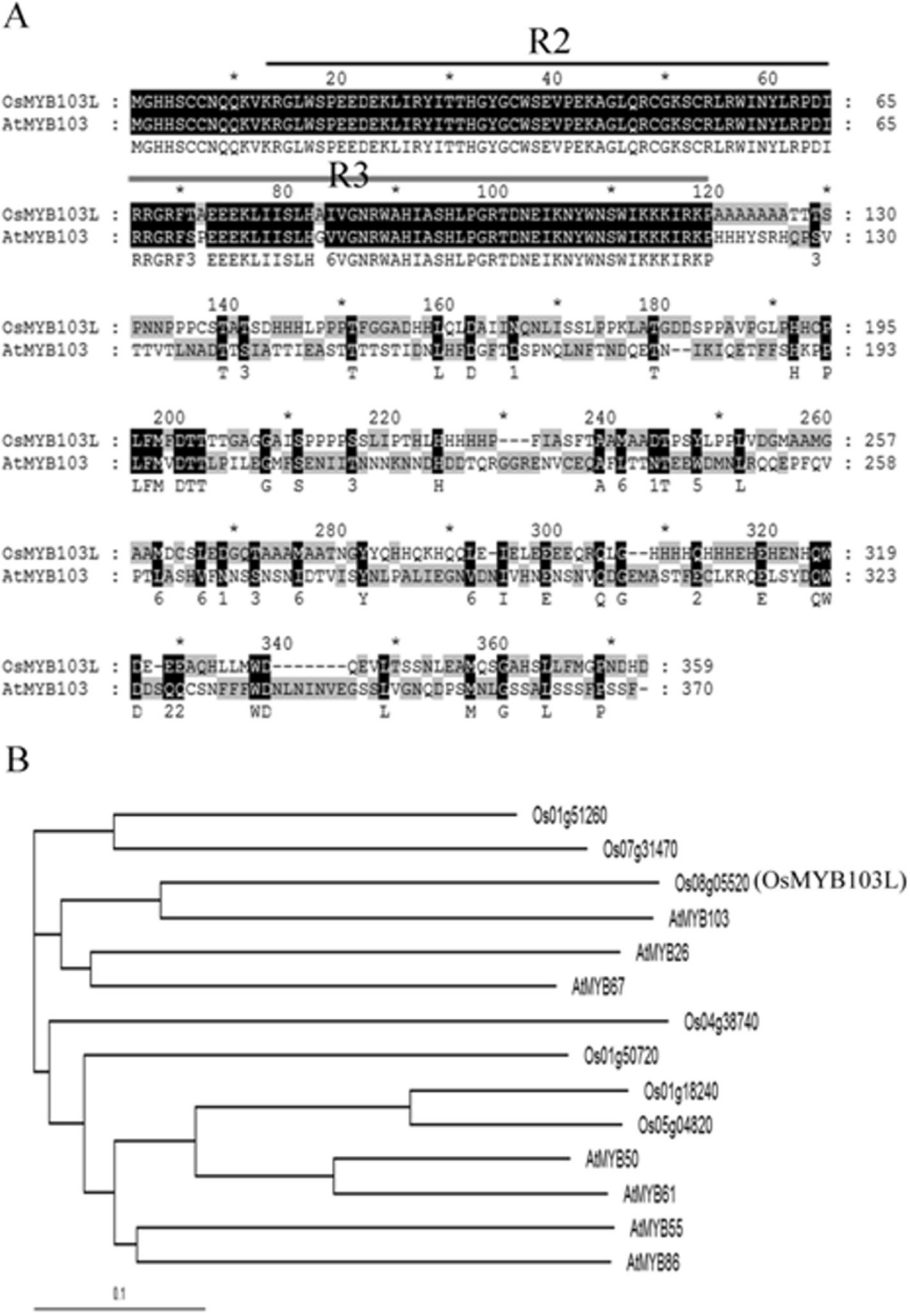
**OsMYB103L (Os08g05520) is an R2R3-MYB transcription factor and has the most sequence homology to AtMYB103 (At1g63910) in *Arabidopsis thaliana*. (A)** Protein sequences alignment of OsMYB103L and AtMYB103. OsMYB103L and AtMYB103 are highly conserved in predicted R2- (black line) and R3-MYB DNA-binding domains (grey line). **(B)** Phylogenetic relationships among OsMYB103L and related R2R3-MYB proteins in rice and *Arabidopsis thaliana*. An unrooted phylogenetic tree was generated with the full-length amino acid sequences.

To discover transcription factors controlling leaf development, we screened the rice lines ectopically expressing rice MYB genes under the control of maize *ubiquitin* promoter. One line overexpressing *LOC_Os08g05520* was selected for further study due to its particular leaf shape, such as upward curling of the leaf blade. According to the rice genome annotation database (http://rice.plantbiology.msu.edu), *LOC_Os08g05520* encodes a putative R2R3-MYB family transcription factor with a length of 359 amino acids and a molecular mass of approximately 40 kD. The Pfam database (http://pfam.sanger.ac.uk/) shows that the deduced protein has two MYB DNA-binding domains (PF00249) at the N-terminus (Figure 1A). As revealed by phylogenetic analysis of the related MYB transcription factors in *Arabidopsis thaliana* and rice, Os08g05520 is closely related to At1g63910 (AtMYB103) [50] (Figure 1B). Protein sequence alignment showed that they are highly conserved in the predicted R2- and R3-MYB DNA-binding domains (Figure 1A). We hereby designated *Os08g05520* as *OsMYB103L* (*Oryza sativa MYB103 Like*).

**Figure 2:**
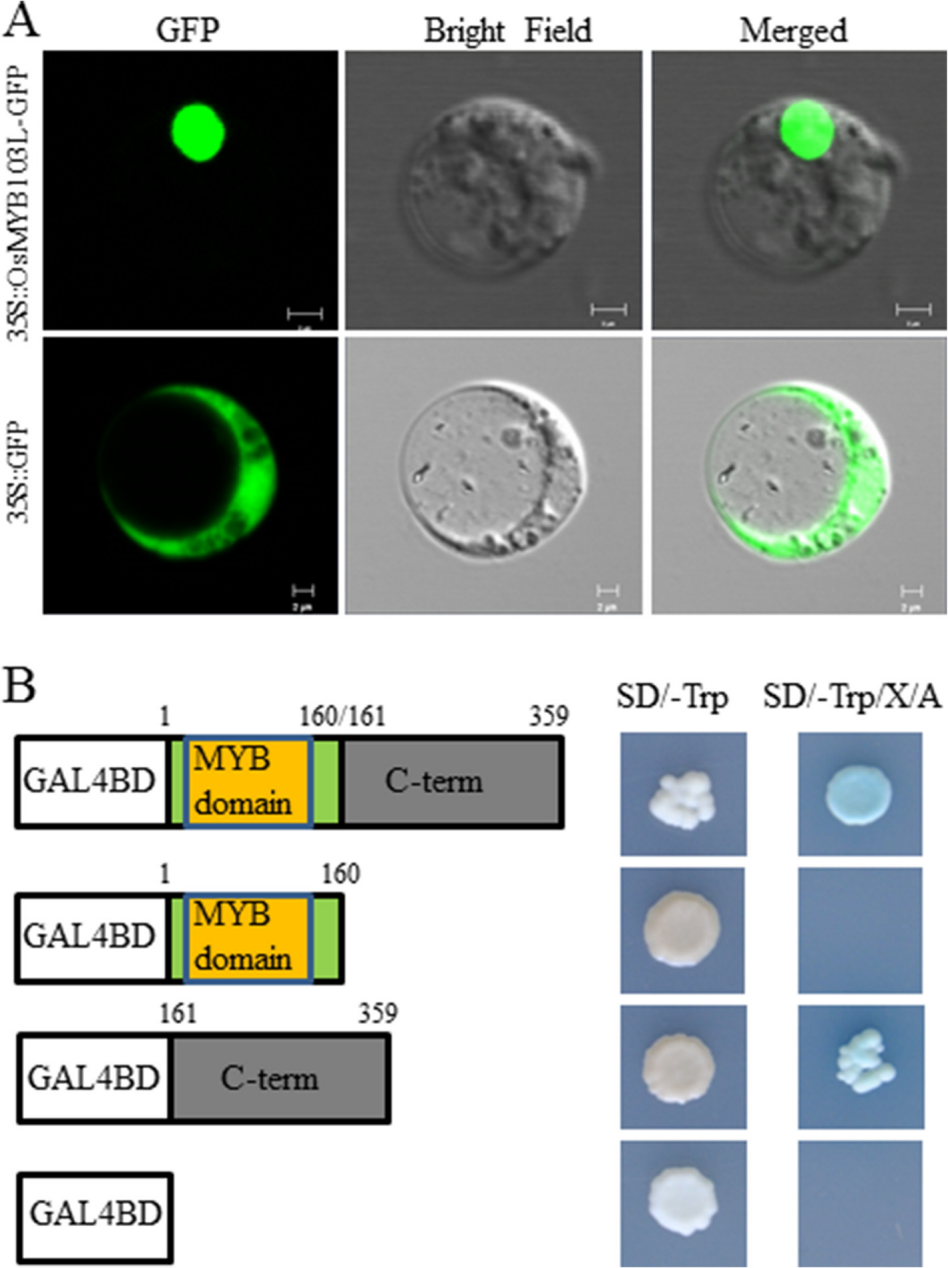
**Subcellular localization and transactivation analysis of OsMYB103L. (A)** Subcellular localization of OsMYB103L. GFP and OsMYB103L-GFP fusion gene under the control of the CaMV35S promoter were expressed transiently in rice protoplasts. Left to right: GFP fluorescence image, transmission image and merged image. Bar = 2 μm. **(B)** Transactivation analysis of different regions of *OsMYB103L* fused with the GAL4 DNA binding domain in yeast. The full-length, N-terminal MYB DNA-binding domain (1–160 amino acids) or the C-terminal putative activation domain (161–359 amino acids) were respectively cloned into pBD-GAL4 vector containing *AUR1-C* and *MEL1* reporter genes and then transformed into yeast host strain Y2HGold. The pBD vector was used as a negative control. The expression of *AUR1-C* confers strong resistance to the highly toxic drug Aureobasidin A (Aba). The activities of α-galactosidase encoded by *MEL-1* were examined by X-α-gal staining.

Nuclear localization is one of the significant features of transcription factors. To determine its subcellular localization, we fused green fluorescent protein (GFP) to the C-terminus of OsMYB103L to produce an OsMYB103L-GFP fusion protein. We monitored the fluorescence of the transiently expressed fusion protein in rice protoplasts and onion (*Allium cepa* L.) epidermis cells. In both cases, the fluorescence signals of the fusion protein were observed predominantly in nuclei (Figure 2A and Additional file 1: Figure S1). While in the GFP alone control, fluorescence signals were observed in nuclei and cytoplasm (Figure 2A and Additional file 1: Figure S1). These results indicate that OsMYB103L is a nuclear-localized protein.

Transcriptional activation is a characteristic feature of many transcription factors. The full-length, N-terminal MYB DNA-binding domain and C-terminal putative activation domain were fused in-frame with the GAL4 DNA binding domain, respectively. We conducted a transcriptional activation analysis in yeast by using the fusion proteins. The analysis showed that the full-length and C-terminal region of OsMYB103L activated the expression of *AUR1-C* and *MEL1* reporter genes in yeast (Figure 2B), indicating that OsMYB103L has the capability of transcriptional activation.

### Expression of *OsMYB103L* varies in different organs

**Figure 3:**
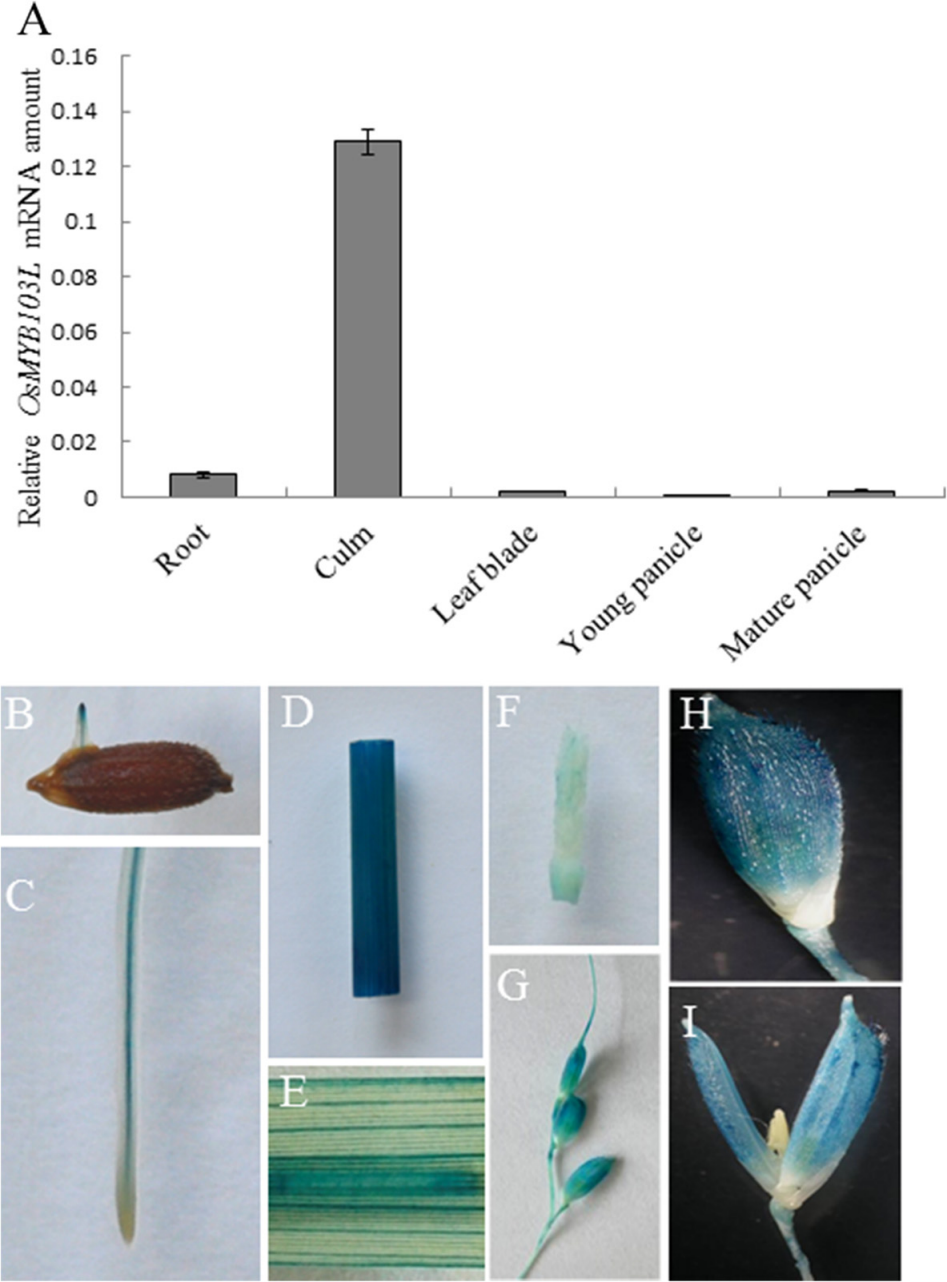
**Expression analysis of *OsMYB103L. *(A)** Expression of *OsMYB103L* in various organs determined by qRT-PCR analysis. Roots were harvested from 2-week-old plants. Culms, leaf blades and mature panicles were harvested from rice plants at heading stage. Young panicles were collected at the 5 cm stage. Rice *ACTIN1* gene was used as internal control. Error bars represent the SD of transcript levels determined from three independent replicates. **(B)** to **(I)** Examination of GUS activity in the transgenic plants expressing *OsMYB103L* pro::GUS. The GUS activity is shown in coleoptiles **(B)**, root **(C)**, culm **(D)**, leaf **(E)**, young panicle **(F)** and mature panicle **(G-I)**. GUS activity was also detected in pedicel, lemma and palea in mature panicle **(H)**, while no GUS activity in sterile lemma, lodicule, pistil and stamen **(I)**.

We determined the expression level of *OsMYB103L* in various rice organs, including root, culm, leave, and panicle using quantitative real-time PCR (qRT-PCR). The expression level of *OsMYB103L* was highest in culm, lower in root, leaf and mature panicle, and lowest in young panicle (Figure 3A).

In addition, the expression pattern of *OsMYB103L* was examined by the *β-glucuronidase* (*GUS*) reporter gene driven by *OsMYB103L* putative promoter in rice plants. Examination of GUS activity in three independent transgenic lines revealed that the GUS signals were stronger in culm and weaker in vascular bundles of coleoptile, root and leaf (Figure 3B to 3I). GUS staining was stronger in mature panicle than young panicle (Figure 3F and 3G). Further examination showed that GUS activity was detectable in pedicel, lemma and palea while no GUS activity was detected in sterile lemma, lodicule, pistil, and stamen in mature panicle (Figure 3H and 3I). These results indicate *OsMYB103L* has diverse expressions in various tissues and organs in rice.

### Overexpression of *OsMYB103L* results in a leaf rolling phenotype

**Figure 4:**
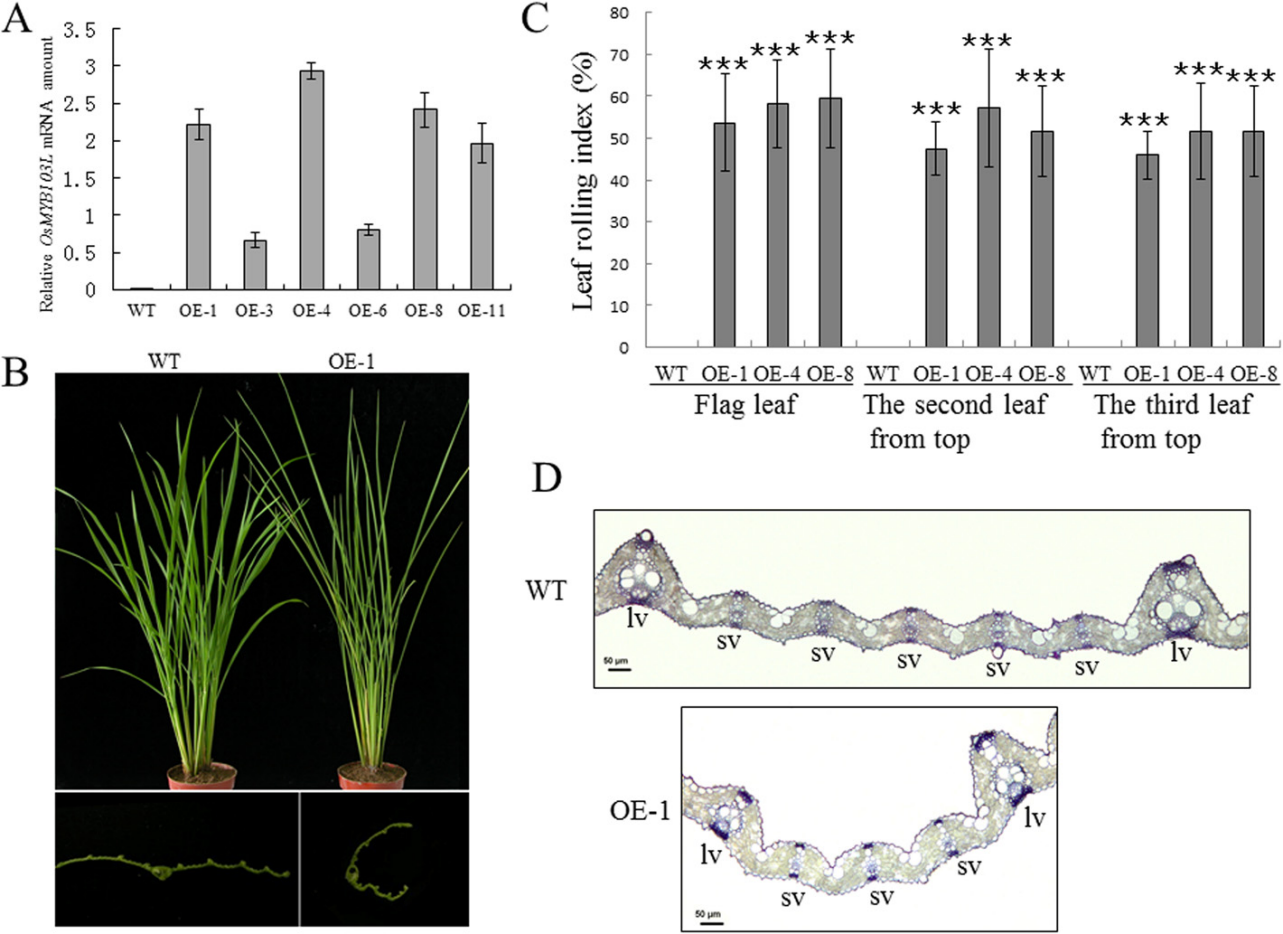
**Phenotypes and molecular analysis of *OsMYB103L *overexpressing plants (OE). (A)***OsMYB103L* expression in OE as determined by qRT-PCR. Rice *ACTIN1* gene was used as internal control. Each column represents an average of three replicates and bars indicate the SDs. **(B)** Morphology of 2-month-old WT and OE leaves. OE showed inward rolled leaves compared to WT (top panel); Cross sections of leaves of WT and OE (bottom panel). Bars = 10 cm (top panel) or 3 mm (bottom panel). **(C)** Statistical analysis of LRIs at the time of flowering for three OE lines of the top three leaf blades (n = 20). Asterisks (***) indicate a significant difference between transgenic plants and WT controls at P < 0.001, by Student’s *t*-test. **(D)** Transverse sections of the flag leaves in the middle part of WT and OE. Shown are regions between two large veins. lv, large vein; sv, small vein. Bars = 50 μm.

Twelve independent transgenic lines overexpressing *OsMYB103L* (OE) were obtained. The expression level of *OsMYB103L* was determined by qRT-PCR (Figure 4A). Three independent transgenic lines (OE-1, OE-4 and OE-8) with the highest expression levels were selected for further study (Figure 4A).The most significant phenotype of OE lines was upward curling of the leaf blades (Figure 4B). This phenotype appeared first at the seedling stage and then maintained through the rest of the plant growth (Figure 4B). The measurement of leaf rolling index (LRI) was performed at the time of flowering. The LRIs of the flag leaf, the second leaf from top, and the third leaf from top were approximately 57%, 52%, and 50%, respectively (Figure 4C). The leaf blade cross-sections did not show significant differences between wild type (WT) and OE in terms of organization of the sclerenchyma and vascular tissues. It suggests that there is no obvious alteration of leaf polarity in transgenic plants. However, in contrast to WT, the leaves of transgenic plants displayed smaller bulliform cells in the rolled regions (Figure 4D).

### Overexpression of *OsMYB103L* affects the expression levels of several genes encoding cellulose synthase (CESA)

**Figure 5:**
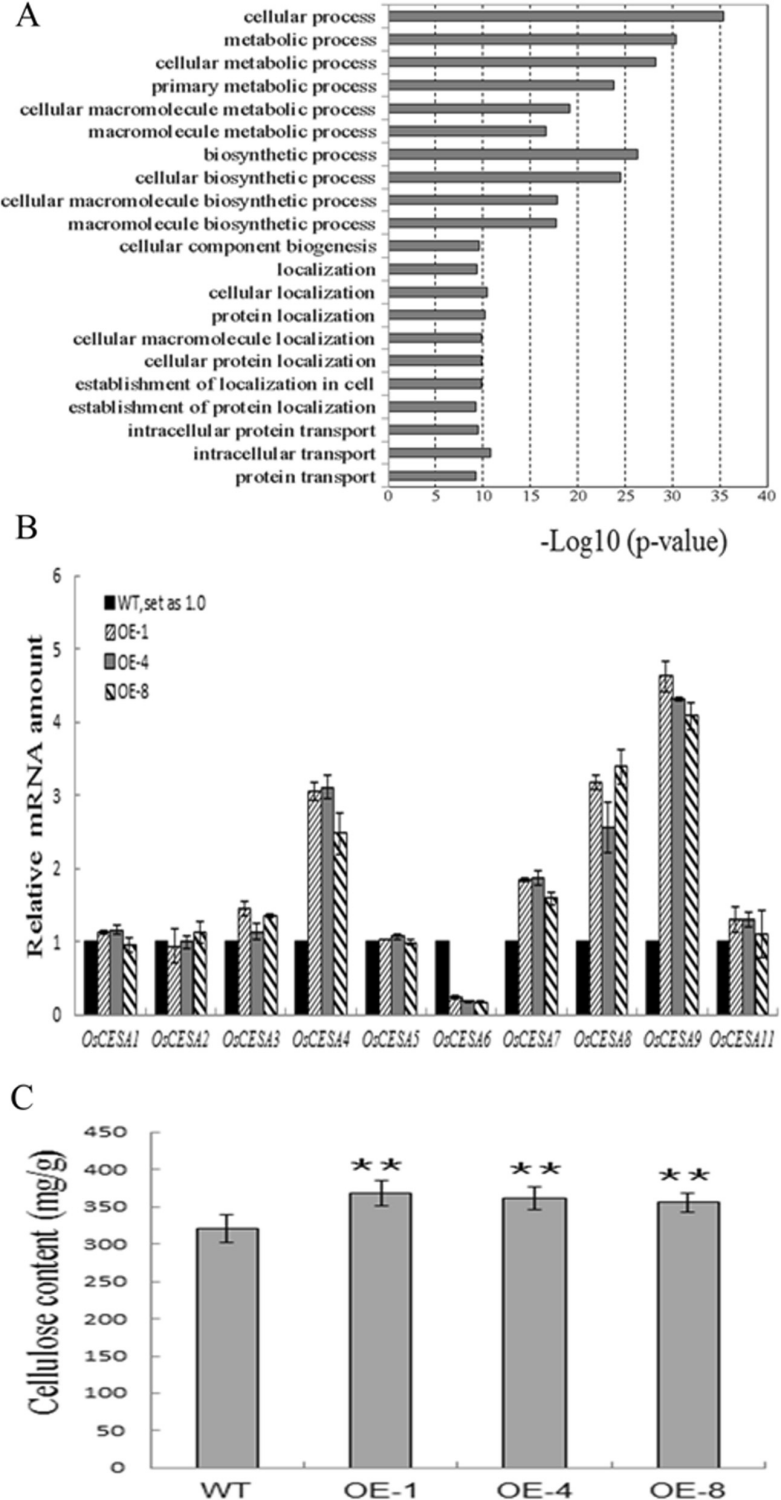
**DGE, qRT-PCR and chemical analysis of wild type (WT) and *OsMYB103L *overexpression (OE) plants. (A)** Main clusters identified by GO enrichment analysis of genes up-regulated in OE compared with WT. In each GO biological process category, the bar indicates the fold enrichment, which is defined as -Log10 (P value). The up-regulated genes are involved in multiple biological processes, such as metabolism, biogenesis, localization and transport. **(B)** Relative expression of rice *CESA* genes in WT and OE was determined by qRT-PCR analysis. The transcript levels of genes were normalized to the levels of *ACTIN1* gene expression. The transcript levels of these genes in WT were arbitrarily set to 1. Values are the mean ± SD of three replicates. **(C)** Measurement of cellulose content in WT and OE. Cellulose content (milligrams per gram of total cell wall residues) of the flag leaf segments from WT and OE plants. The error bars were obtained from five measurements. Asterisks (**) indicate a significant difference between transgenic plants and WT controls at P < 0.01, by Student’s *t*-test.

To explore the possible molecular mechanism of OsMYB103L’s effects on rice leaf development, we examined the expression profile in OE-1 and WT leaf blades using Digital Gene Expression profiling analysis (DGE). In all, 3,036 annotated genes showed expressions with more than twofold difference between OE-1 and WT, of which 2,026 genes were up-regulated and 1,010 genes were down-regulated (Additional file 1: Figure S2 and Additional file 2: Table S1). To obtain an ontological profile of the up- or down-regulated genes, ontological terms were assigned, and the enrichment significance was analyzed by AgriGO [51]. Twenty-one Gene Ontology (GO) terms were significantly enriched in the up-regulated gene set, showing that up-regulated genes were involved in multiple biological processes, such as metabolism, biogenesis, localization, and cellular processes (Figure 5A and Additional file 1: Figure S3). Meanwhile, we noticed that two GO terms, cellulose metabolic process (GO:0030243) and cellulose biosynthetic process (GO:0030244), were possibly related to the leaf rolling trait as reported in *rl14* mutant [2] (Additional file 1: Figure S3). Therefore, we compared the expression levels of the rice *CESA* genes [52]. Our DGE data showed that *OsCESA1*, *OsCESA4*, *OsCESA7, OsCESA8*, *OsCESA9,* and *OsCESA11* were up-regulated while *OsCESA5* and *OsCESA6* were down-regulated (Additional file 1: Table S2). We verified the expressions by qRT-PCR for these *CESA* genes in three independent lines (OE-1, OE-4 and OE-8). Four *CESA* genes, *OsCESA4*, *OsCESA7*, *OsCESA8*, and *OsCESA9*, showed significantly increased transcript levels; while one gene, *OsCESA6*, was down-regulated in the OE plants (Figure 5B). The qRT-PCR analysis was largely consistent with the DGE analysis except three genes, *OsCESA1* and *OsCESA11* of which, due to their relatively low Log_2_Ratio in DGE data. CESAs are proteins responsible for cellulose synthesis in plants [52-54], thus alteration of *CESA* expression may change the cellulose content. Therefore, we measured cellulose content in leaf blades of WT and OE plants. The results showed that OE leaf blades had 13% higher cellulose content compared to WT (Figure 5C). These results suggest that *OsMYB103L* regulates *CESA* gene expression and may consequently influence cellulose synthesis in rice.

### Knockdown of *OsMYB103L* decreases the mechanical strength and cellulose content of leaves

**Figure 6:**
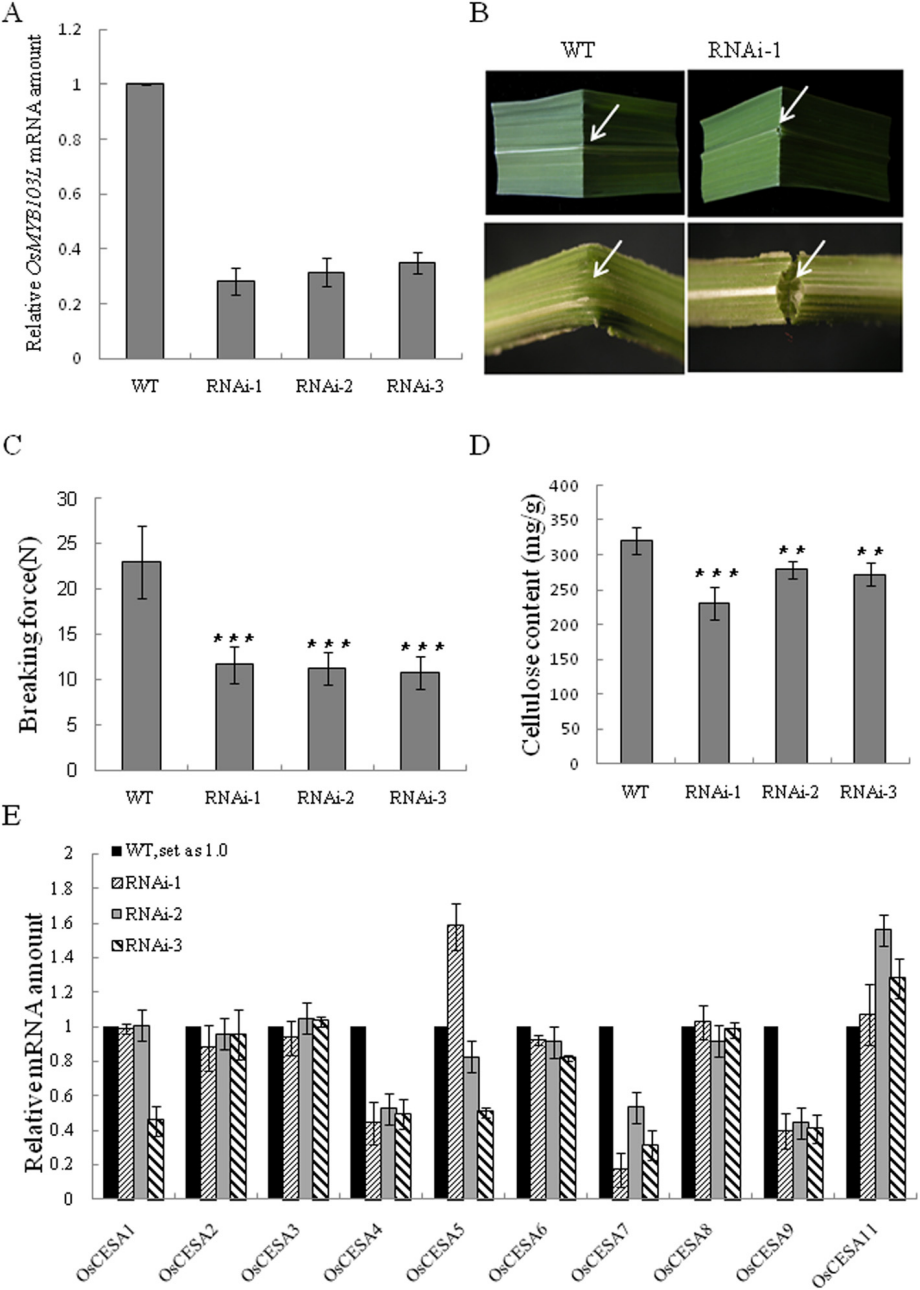
**Down-regulation of *OsMYB103L *decreases mechanical strength and affected cellulose biosynthesis. (A)** Quantitative RT-PCR analysis for RNAi transgenic plants. The transcript levels of *OsMYB103L* in WT were arbitrarily set to 1. Values are the mean ± SD of three replicates. **(B)** An easily broken *OsMYB103L* RNAi-1 leaves, as indicated by the arrow. **(C)** The force required to break WT and *OsMYB103L* RNAi lines flag leaves. Error bar represents SD (n =10 in each measurement). **(D)** Comparison of cellulose content between the wild type and *OsMYB103L* RNAi lines flag leaves. Error bar represents SD (n = 5 in each measurement). **(E)** Relative expression of rice *cellulose synthase* (*CESA*) genes in wild type (WT) and *OsMYB103L* RNAi plants was determined by qRT-PCR analysis. The transcript levels of genes were normalized to the levels of *ACTIN1* gene expression. The transcript levels of these genes in wild type were arbitrarily set to 1. Values are the mean ± SD of three replicates. Asterisks (** or ***) indicate a significant difference between transgenic plants and WT controls at P < 0.01 or 0.001, respectively, by Student’s *t*-test.

To explore the intrinsic function of *OsMYB103L*, we analyzed the phenotypes of mutants with decreased expression levels. Since the loss-of-function mutants were not available, we created RNAi-knockdown plants for *OsMYB103L.* Six independent RNAi transgenic lines were obtained. Quantitative RT-PCR analyses showed that the expression level of *OsMYB103L* was significantly decreased in leaves of the RNAi lines compared with that of WT (Figure 6A). There were no obvious morphological changes in RNAi lines. However, in the heading stage of the RNAi plants, we noticed that the leaves were brittle. When the leaf blade was bent, WT leaves were flexible while the leaves of RNAi lines were broken easily, especially in the midribs (Figure 6B). The force required to break the leaf blades was 50% less for the RNAi lines than that for WT (Figure 6C). The weakened mechanical strength may relate to the altered cellulose content in leaf blades. We measured the cellulose content in leaf blades of WT and RNAi plants. Compared with WT, the levels of cellulose content in RNAi lines were significantly decreased (Figure 6D). Therefore, the decreased mechanical strength of RNAi plants might result from the decreased cellulose content. We determined the expression levels of ten *CESA* genes by qRT-PCR, and found that three *CESA* genes, *OsCESA4*, *OsCESA7*, and *OsCESA9* were significantly down-regulated in the RNAi lines (Figure 6E). These results suggest that the down-regulated CESA genes in *OsMYB103L* RNAi plants may lead to a decreased level of cellulose content and the weakened mechanical strength of transgenic leaf blades.

## Discussion

By screening phenotypes of a transgenic rice population overexpressing rice genes in MYB family, we identified a rice leaf rolling-related *R2R3-MYB* gene, *OsMYB103L*. We showed its activity of transcriptional activation and described its influence on leaf rolling. We further showed that the altered expression of *OsMYB103L* resulted in changes in *CESA* gene expression, cellulose content, and mechanical strength in rice leaf. These results suggest that *OsMYB103L* may have important biological functions in rice leaf development.

Leaf rolling is one of the frequently observed phenotypes in rice and other cereals. It is one of the important agronomic traits for high yield rice varieties [6-9]. Although many cultivars and mutants in rice show several types of leaf rolling phenotypes and a number of leaf rolling related genes have been identified [1-3,19,20,55], the mechanisms underlying the rolling phenomena remain to be elucidated. It is generally realized that two major factors can influence leaf rolling. The first one is the establishment of cell polarity and differentiation, and the second is physiological factors, such as osmotic pressure or turgidity in bulliform cells [1,2,56,57]. Previous studies on mutants defective in leaf development in *Arabidopsis thaliana*, maize, and rice have shown that leaf rolling is related to the defective development of the leaf polarity along the adaxial–abaxial axis [1,58]. However, we did not observe obvious alterations in leaf polarity in the *OsMYB103L* overexpressing plants by histological analysis (Figure 4D) and SEM observations (Additional file 1: Figure S4). Instead, we found that the bulliform cells in the rolled regions of OE leaves were smaller compared with that of WT leaves (Figure 4D). Several investigations have shown that bulliform cells are involved in leaf rolling [2,59]. Loss of turgor pressure in bulliform cells could lead to leaf “roll up” during water stress [56]. Here the observed abnormal bulliform cells in the leaves of *OsMYB103L* overexpressing plants are consistent with those described in a number of known rolled leaf mutants [2,20]. For instance, c*ow1* and *rl14* mutants showed rolled leaves with serious shrinkage and smaller bulliform cells, but with no obvious defects in leaf polarity [2,20]. Therefore, our results suggest that the abnormal bulliform cells, rather than defects in leaf polarity, may account for the rolled leaf phenotype in *OsMYB103L* overexpressing plants to some extent.

Secondary cell walls constitute skeletal frameworks and furnish the plant body with mechanical strength [60,61]. Molecular and genetic studies in the model plant, *Arabidopsis thaliana*, revealed that a group of secondary wall-associated NAC domain (SWN) transcription factors are master switches regulating a cascade of down-stream transcription factors, such as MYB transcription factors that leads to the activation of the secondary wall biosynthetic program [62,63]. It has been reported that *AtMYB103* (*At1g63910*) is directly activated by the master switches, SWNs [62,63]. In *Arabidopsis thaliana*, overexpression of *R2R3-MYB* gene *AtMYB103* led to a noticeable increase in the secondary wall thickness, while its dominant repression caused reduction in secondary wall thickness of both xylary fibers and interfascicular fibers [50,64]. It has been proposed that AtMYB103 has the potential to induce the expression of genes involved in cellulose biosynthesis [50]. The transcriptional network regulating secondary cell wall biosynthesis may be evolutionarily conserved in plants, however, little is known about the detailed regulatory programs in grass species, such as rice [65].

Based on our analyses on the *OsMYB103L* and its encoded protein, we deduced that *OsMYB103L* might be an ortholog of *AtMYB103* in rice. We wondered whether it has the conserved functions in rice, a model plant in monocotyledon, and addressed this question by reverse genetics, including overexpression and RNAi-knockdown approaches. Our study showed that overexpression of *OsMYB103L* resulted in rolled leaves and knockdown of *OsMYB103L* led to brittle leaves (Figures 4B and 6B). Further analyses disclosed that the expression of four rice *CESA* genes, *OsCESA4*, *OsCESA7*, *OsCESA8*, and *OsCESA9*, was increased significantly in transgenic overexpression rice plants (Figure 5B and Additional file 2: Table S1) whereas three of these *CESA* genes, *OsCESA4*, *OsCESA7*, and *OsCESA9*, were significantly down-regulated in the RNAi lines (Figure 6E). OsMYB103L is a putative R2R3 MYB transcription factor, which is expected to regulate gene expression by binding to the promoters of its downstream genes. Putative MYB binding sequences (MBSs) can be identified by using the tools described in PLACE (A Database of Plant Cis-acting Regulatory DNA Elements; http://www.dna.affrc.go.jp/PLACE/) [66]. We did find two and even more CNGTTR sites, the putative MBSs, in the respective promoter regions of these *CESA* genes (Additional file 1: Figure S5). Therefore, we hypothesized that these *CESA* genes would be the targets of OsMYB103L. Further investigation, such as chromatin immunoprecipitation (ChIP) and electrophoretic mobility shift assay (EMSA), could be carried out to prove it.

In higher plants, CESAs were characterized as catalytic subunits of cellulose synthase complexes [67,68]. In this study, our results showed that the cellulose content of mature leaves was higher in overexpression lines than that in WT (Figure 5C) while the *OsMYB103L* RNAi lines had decreased cellulose content in the leaf blades (Figure 6D). The results indicate that OsMYB103L may regulate the expression levels of *CESA* genes, which could subsequently alter the cellulose content. However, we also noticed that the contents of cellulose were not positively correlated with the expression levels of *OsMYB103L* in transgenic plants, especially in the overexpression lines. Plant *CESA* genes are members of multigene families [69]. *Arabidopsis* has 10 CESA isoforms which can be classified into six orthologous groups having non-redundant functions in cellulose synthesis as revealed by mutational analysis [53]. Rice also has 10 CESA isoforms, of which OsCESA4, OsCESA7 and OsCESA9 were showed non-redundant functions in cellulose synthesis specifically in secondary walls [52,70]. Based on these, we speculate that most rice CESAs might also play distinctive roles in cellulose synthesis. Some particular *CESA* gene expression induction may not lead to significant change of cellulose content. Therefore, changed expressions of several *CESAs* in *OsMYB103L* transgenic lines may not induce obviously changes in cellulose content of the transgenic plants.

In addition, our study demonstrated that *OsMYB103L* RNAi-knockdown plants possessed brittle leaves with obviously decreased mechanical strength and reduced cellulose content in the leaf blades (Figure 6B- 6D). Of note, reduced cellulose content and inferior mechanical strength are the common phenotypes of *CESA* mutants, such as *irx1* to *irx3* in *Arabidopsis* and *brittle culm13* (*bc13*) in rice [70-72]. Thus, we wondered whether mechanical strength was elevated due to the increased cellulose in *OsMYB103L* overexpression plants. Therefore, we also measured the mechanical strength of the overexpression lines of *OsMYB103L*. Nevertheless, the leaves of overexpression plants did not show statistically significant changes in mechanical strength compared to wild type (Additional file 1: Figure S6). Mechanical strength is a complex trait, which can be affected by many factors [73,74]. Perhaps, in our case, the increase of cellulose contents was not enough to cause the change of mechanical strength in *OsMYB103L* overexpression plants.

Moderate leaf rolling has been regarded as one of the important agronomic traits in high yield rice cultivars [6-9], and selection of this trait has been applied for rice breeding directly or indirectly in China [5,6]. One of the successful examples is the breeding of super-hybrid rice, Liang-You-Pei-Jiu (LYP9) [75,76]. LYP9 has moderate xrolling leaves and is widely cultivated throughout southern China due to its high yield and seed quality. The successful cultivation of LYP9 is therefore taken as an example for improving crop yield and quality using the rolled leaf trait [77]. Therefore, identification of genes that regulate leaf rolling would be beneficial to breeding. In this study, we report that overexpression of *OsMYB103L* in rice results in leaf rolling, which suggests that *OsMYB103L* could be a potential target for genetic manipulation of leaf shape and plant architecture in rice. In addition, leaf brittleness is an important agronomic trait for rice and other crops since it affects not only grain production but also the utilization of cereal straw as animal forage [74]. Here, we found that down-regulation of the expression level of *OsMYB103L* decreases mechanical strength in rice plants, suggesting that *OsMYB103L* could be engineered for favorable leaf brittleness in rice, as well as in other cereal and forage crops.

## Conclusions

In this study, an R2R3 MYB transcription factor, *OsMYB103L*, has been identified in rice. We show that overexpression of *OsMYB103L* in rice results in leaf rolling, increased cellulose content, and elevated expression of genes encoding cellulose synthase (CESA). Besides, the RNA interference (RNAi)-knockdown of *OsMYB103L* leads to a weakened mechanical strength and decreased cellulose content in leaves. Moreover, the expression levels of several *CESA* genes are up-regulated in *OsMYB103L* overexpression lines while down-regulated in *OsMYB103L*-knockdown lines. Collectively, this work demonstrates that *OsMYB103L* impacts leaf shape, cellulose synthesis, and mechanical strength in rice.

## Methods

### Plant materials and growth conditions

Rice (*Oryza sativa* L.) subspecies *indica* cultivar Kasalath and *japonica* cultivar Nipponbare were used for analysis. Rice seeds from control wild type and transgenic lines were immersed in water for 2 days and grown in a soil seed bed for approximately 30 days, and then the seedlings were transplanted to the field and/or greenhouse for further analysis. Plants maintained in greenhouses were grown under standard conditions at 28°C with 16 h of light. The fields were located in the Experimental Stations (Beijing and Hainan) of the Institute of Genetics and Developmental Biology, Chinese Academy of Sciences, Beijing, China.

### Bioinformatics analysis

A search for OsMYB103L homologs was performed using the National Center for Biotechnology Information (NCBI) BLAST server (http://blast.ncbi.nlm.nih.gov/Blast.cgi). Domain prediction was performed using the Pfam database (http://pfam.sanger.ac.uk) and the NCBI Conserved Domains database (http://www.ncbi.nlm.nih.gov/Structure/cdd/cdd.shtml). Alignment was performed with ClustalW using default setting (http://clustalw.ddbj.nig.ac.jp/). Unrooted neighbor-joining phylogenetic tree was constructed using the neighbor-joining algorithm with PHYLIP version 3.572c [78]. Bootstrap analysis was carried out with 1,000 replicates. TreeView was used to generate the graphical output [79].

### Subcellular localization

For detection of subcellular localization of OsMYB103L, the open reading frame (ORF) of *OsMYB103L* was amplified from the FL-cDNA clone (AK111808) and then was cloned into vector pEZR(K)-LN to create the 35S:OsMYB103L-GFP vector. The 35S: OsMYB103-GFP and empty vector (pEZR(K)-LN) were transformed into rice protoplasts by polyethylene glycol-mediated transformation method [80] or onion epidermis by particle bombardment using a PDS-1000/He biolistic particle delivery system (Bio-Rad, California, USA), respectively. Subcellular distribution of the OsMYB103L protein was visualized by fluorescent confocal microscopy (Leica TCS SP5, California, USA).

### Transactivation assay in yeast cells

The yeast strain Y2HGold, containing the *AUR1-C* and *MEL1* reporter genes with GAL4 binding elements in the promoters, respectively, was used as an assay system (Clontech, USA). The expression of *AUR1-C* confers strong resistance to the highly toxic drug Aureobasidin A (Aba). The activities of α-galactosidase which *MEL-1* encoded were examined by X-α-gal staining. The full-length, N-terminal MYB DNA-binding domain (1–160 Amino acids) or the C-terminal putative activation domain (161–359 Amino acids) were respectively cloned into pBD-GAL4 vector. The pBD vector was used as a negative control. All these plasmids were individually introduced into cells of yeast strain Y2HGold. The yeast transformants were grown on SD medium in the absence of tryptophan (SD/-Trp) for 2–3 days at 30°C. Transferred yeast cells were also grown on SD/-Trp in the presence of Aba and X-α-gal for 2–3 days at 30°C to monitor the generation of blue color.

### Promoter-GUS analysis

The promoter of the *OsMYB103L* (about 1.4 kb upstream of ATG) was amplified from the rice genomic DNA (cv. Nipponbare) using the primers 5′-GGATCCACTCCTGCGAGGCTCTGATACG-3′ and 5′-CCATGGATGCACTCT-AGATATCACTG-3′. Then, the DNA fragment was cloned into the pCAMBIA1301 (CAMBIA, Australia) vector resulting in fusion of the promoter and the GUS reporter gene. The *ProOsMYB103: GUS* construct was transformed into wild type rice cultivar Kasalath. About 20 independent transgenic lines were obtained after screening. GUS staining was performed according to the method of Jefferson *et al*. [81].

### Binary vectors construction and rice transformation

For the overexpression construct of *OsMYB103L*, gene-specific primers, 5′-*GGATCC*TGCTAGCAGCTAGATCAAG-3′ and 5′-*ACTAGT*GTCATCCTCCTG-TGTTTATT-3′ (*Bam*H I and *Spe* I sites italicized) were used to amplify the coding sequence of *OsMYB103L* using the FL-cDNA clone (AK111808) as template. The FL-cDNA clone was from the National Institute of Agrobiological Sciences, Japan [78]. The sequencing-confirmed PCR fragment was cloned into the vector pTCK303 [82] to create the overexpression vector of *OsMYB103L* driven by a maize *ubiquitin* promoter*.*

For the RNA interference (RNAi) construct of *OsMYB103L*, a fragment of approximately 325 bp was amplified from *OsMYB103L* with two primers, 5′-*GGTACCACTAGT*CCGTGAAGCTGGCGATGAAC-3 (*Kpn* I and *Spe* I sites italicized) and 5′-*GGATCCGAGCTC*CACGACGAGCCCGAACAAC-3′ (*Bam*H I and *Sac* I sites italicized). The sequencing-confirmed PCR fragment was cloned into the vector pTCK303 as previously described [82] to create the RNAi vector of *OsMYB103L* driven by a maize *ubiquitin* promoter*.*

These constructs were transferred to *Agrobacterium tumefaciens* strain LBA4404 by electroporation, and were transformed into an *indica* rice cv. Kasalath, respectively, according to the method of Hiei *et al*. [83].

### RNA extraction and qRT-PCR analysis

Total RNA was extracted from different tissues using Trizol reagent (Invitrogen, California, USA) according to the manufacturer’s protocol. After treatment with DNase to remove genomic DNA contamination, the first strand of cDNA was synthesized by M-MLV reverse transcriptase (Promega, USA). Relative quantification of gene expression by real-time PCR was performed on a Bio-Rad Chromo 4 Real Time PCR System (Bio-Rad, USA) with TransStart SYBR Green qPCR SuperMix Kit (TransGen, Beijing, China). The housekeeping gene *ACTIN1* was used as an internal control for normalization of RNA samples. Three replicates were carried out for each gene and each analysis was biologically repeated at least twice. Gene-specific primers are shown in Additional file 1: Table S3 line.

### Measurement of the leaf rolling index (LRI)

To determine the leaf rolling index (LRI), two measurements were taken, Lw (Expand the leaf blade and determine the greatest width of the leaf blade) and Ln (Measure the natural distance of the leaf blade margins at the same site). LRI was calculated as LRI (%) = (Lw-Ln)/Lw × 100 [3]. Data were collected on the flag (uppermost), second and third leaves on twenty individual plants at the time of flowering.

### Cellulose measurement

Mature leaves were cut into pieces and ground into powder under liquid nitrogen. The materials were then extracted twice with 70% ethanol at 70°C for 1 h. Cell wall materials were dried under vacuum for cellulose content measurement [84]. Whatman 3MM filter paper was used to establish a standard curve for quantification of cellulose. The anthrone reagent was used to determine the cellulose content.

### Breaking force test

To examine the mechanical strength in leaf blade, the leaf blades of flag leaves from developmentally matched transgenic rice plants and wild type plants were collected and immediately used for measurement. The forces that break the samples at leaf blades were measured with a digital force/length tester (5848 Microtester; Instron, USA).

### Scanning electron microscopy (SEM) observation

Samples were fixed in 2.5% glutaraldehyde solution. Fixed samples were dehydrated with gradual ethanol series, dried by critical-point drying method using liquid carbon dioxide (Model HCP-2, Hitachi, Tokyo), gold-coated with an Edwards E-1010 ion sputter coater (Hitachi, Tokyo), and then observed using a S-3000 N variable pressure scanning electron microscope (Hitachi, Tokyo).

### Microscopy

For light microscopic analysis, the samples were fixed in 2.5% glutaraldehyde solution, and then dehydrated through a graded acetone series. The samples were embedded in Epon812 (SPI CHEM) and polymerized at 70°C. Sections were cut with a microtome (Leica RM2265, Germany) and stained with 0.25% toluidine blue, and finally observed with a microscope (OLYMPUS BX41, Japan).

### Digital gene expression profiling (DGE) analysis

For digital gene expression analysis, transgenic overexpressor (OE-1) and wild type flag leaves were harvested for RNA extraction using TRIzol (Invitrogen) as described by the manufacturer. The digital expression analysis was performed by the Beijing Genomics Institute (http://www.genomics.cn) using the standardized procedure. The main reagents and supplies are Illumina Gene Expression Sample Prep Kit and Illumina Sequencing Chip (flowcell), and the main instruments are Illumina Cluster Station and Illumina HiSeqTM 2000 System. All clean tags were mapped to the reference sequences (ftp://ftp.plantbiology.msu.edu/pub/data/Eukaryotic_Projects/o_sativa/annotation_Dbs/pseudomolecules/version_6.1/all.dir/all.cDNA) and only 1 bp mismatch is considered. Clean tags mapped to reference sequences from multiple genes were filtered. Remainder clean tags were designed as unambiguous clean tags. The number of unambiguous clean tags for each gene was calculated and then normalized to TPM (number of transcripts per million clean tags).

The original data set is deposited in the National Institutes of Health Gene Expression Omnibus database under accession number GSE52394.

### Accession numbers

Sequence data used in this manuscript can be found in the Arabidopsis Information Resource (TAIR, http://www.arabidopsis.org) and the rice genome annotation database (http://rice.plantbiology.msu.edu) under the following accession numbers: *AtMYB103* (*At1g63910*), *AtMYB26* (*At3g13890*), *AtMYB50* (*At1g57560*), *AtMYB55* (*At4g01680*), *AtMYB61* (*At1g09540*), *AtMYB67* (*At3g12720*), *AtMYB86* (*At5g26660*), *OsMYB103L* (*Os08g05520*), *Os01g51260*, *Os01g18240*, *Os05g04820*, *Os07g31470*, *Os01g50720*, *Os04g38740*, *OsActin1* (*Os03g50890*), *OsCESA1* (*Os05g08370*), *OsCESA2* (*Os03g59340*), *OsCESA3* (*Os07g24190*), *OsCESA4* (*Os01g54620*), *OsCESA5* (*Os03g62090*), *OsCESA6* (*Os07g14850*), *OsCESA7* (*Os10g32980*), *OsCESA8* (*Os07g10770*), *OsCESA9* (*Os09g25490*), *OsCESA11* (*Os06g39970*).

## Competing interests

The authors declare that they have no competing interests.

## Authors’ contributions

YC and DL carried out the most of molecular genetic studies, data analysis and drafted the manuscript. YC, XLiu, and XLi carried out the rice transformation. CJ participated in microscopy analysis. LH participated in bioinformatics analysis. XZ participated in phenotype analysis. CC and ZC participated in the data analysis. LZ and DL conceived of the study, and participated in its design, coordination and drafted the manuscript. All authors read and approved the final manuscript.

## Supplementary Material

Additional file 1: Figure S1 OsMYB103L-GFP is located to nucleus in onion epidermal cells. **Figure S2.** Number of differentially expressed genes in Digital Gene Expression profiling analysis (DGE) between wild type and *OsMYB103L* overexpressing plants. **Figure S3.** GO enrichment analysis of genes up-regulated in wild type and *OsMYB103L* overexpressing plants. **Figure S4.** Scanning electron micrographs (SEM) analysis. **Figure S5.** Schematic diagrams of the promoter regions of *OsCESA* genes. **Figure S6.** The force required to break flag leaves in wild type and *OsMYB103L* overexpression plants. **Table S2.** Expression levels of *CESA* genes in DGE analysis between wild type (WT) and *OsMYB103L* overexpressing plants (OE-1). **Table S3.** Primers of qRT-PCR used in this article. Click here for file

Additional file 2: Table S1 Genes that up- or down-regulated two-fold in DGE data of *OsMYB103L* overexpressing plants compared to that of the wild type (Excel File). Click here for file
