# Supplementary material for: OsMYB103L, an R2R3-MYB transcription factor, influences leaf rolling and mechanical strength in rice (Oryza sativa L.)
Source: BMC Plant Biol. 2014 Jun 6;14:158. doi: 10.1186/1471-2229-14-158 (PMC4062502; doi:10.1186/1471-2229-14-158)
Supplement: Additional file 1: Figure S1 — OsMYB103L-GFP is located to nucleus in onion epidermal cells. Figure S2. Number of differentially expressed genes in Digital Gene Expression profiling analysis (DGE) between wild type and OsMYB103L overexpressing plants. Figure S3. GO enrichment analysis of genes up-regulated in wild type and OsMYB103L overexpressing plants. Figure S4. Scanning electron micrographs (SEM) analysis. Figure S5. Schematic diagrams of the promoter regions of OsCESA genes. Figure S6. The force required to break flag leaves in wild type and OsMYB103L overexpression plants. Table S2. Expression levels of CESA genes in DGE analysis between wild type (WT) and OsMYB103L overexpressing plants (OE-1). Table S3. Primers of qRT-PCR used in this article. [file 1471-2229-14-158-S1.doc]

**Additional file1**

**Legends for additional file1**

**Figure S1.** OsMYB103L-GFP is located to the nucleus in onion epidermal cells. GFP and the OsMYB103L-GFP fusion gene under the control of the CaMV 35S promoter were expressed transiently in onion epidermal cells. Left to right: GFP fluorescence image, transmission image, and merged image.

**Figure S2.** Number of differentially expressed genes in Digital Gene Expression profiling analysis (DGE) between wild type and *OsMYB103L* overexpressing plants. A total of 3036 annotated genes showed altered expression, of which 2026 genes were up-regulated and 1010 genes were down-regulated in *OsMYB103L* overexpressing plants compared to those in wild type.

**Figure S3.** GO enrichment analysis of genes up-regulated in wild type and *OsMYB103L* overexpressing plants. The red rectangle indicates two pathways possibly related to rolled leaf as reported in *rl14* mutant .

**Figure S4.** Scanning electron micrographs (SEM) analysis. SEM analysis of *OsMYB103L* overexpression lines (OE) and wild type (WT) rice plants showed that there was no obvious alteration in leaf polarity. The result showed that there was no obvious difference at the adaxial or abaxial surface between WT and OE. Bars = 50 mm.

**Figure S5.** Schematic diagrams of the promoter regions of *OsCESA* genes. Black lines represent the promoter region of the *OsCESA* genes. The MYB binding sequences (MBSs) were predicted using PLACE software (http://www.dna.affrc.go.jp/PLACE/; Higo et al., 1999). Black boxes on the line represent the putative MBSs. Numbers above indicate the distance away from the ATG. The translational start sites (ATG) were shown as +1 (Scale bars: 300 bp).

**Figure S6.** The force required to break flag leaves in wild type and *OsMYB103L* overexpression plants. Error bar represents SD (n =10 in each measurement).

**Table S2.** Expression levels of *CESA* genes in DGE analysis between wild type (WT) and *OsMYB103L* overexpressing plants (OE-1).

**Table S3.** Primers of qRT-PCR used in this article.

**Supplementary Figures**


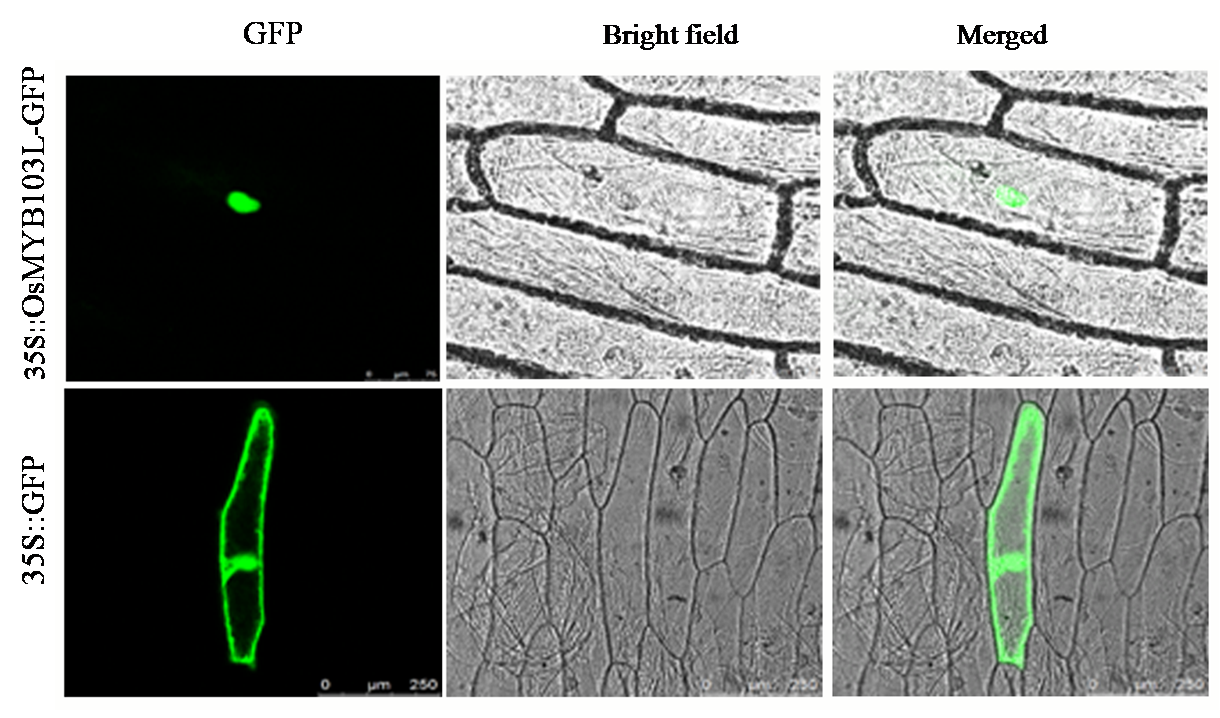


**Figure S1.**


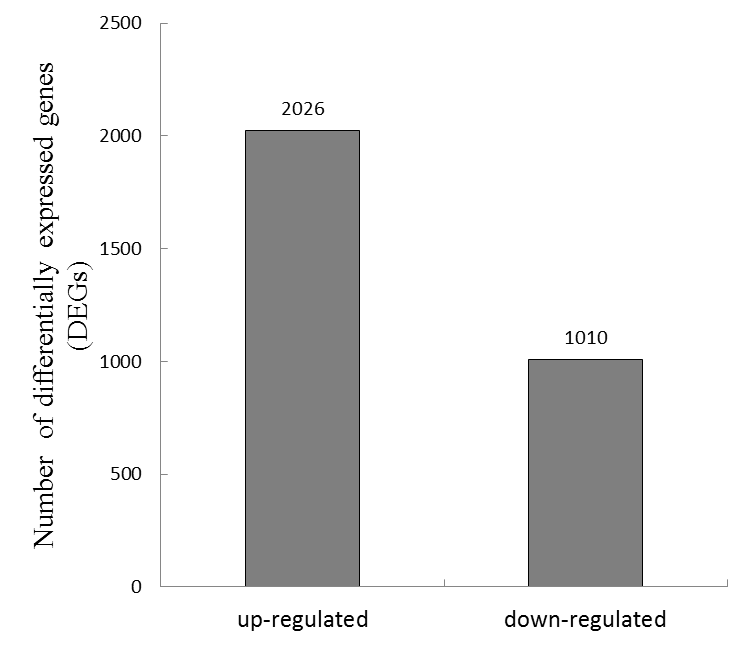


**Figure S2.**


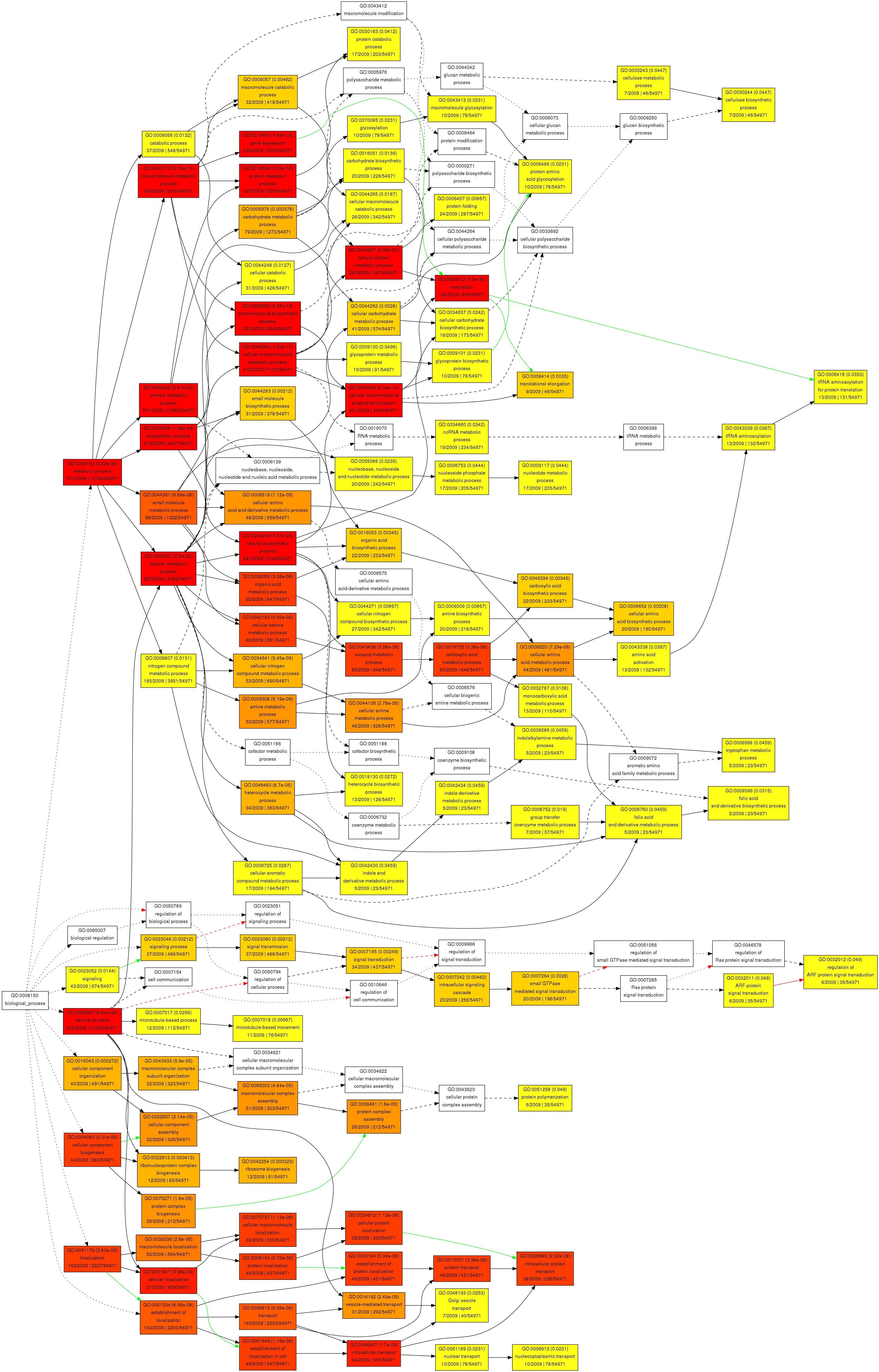


**Figure S3.**


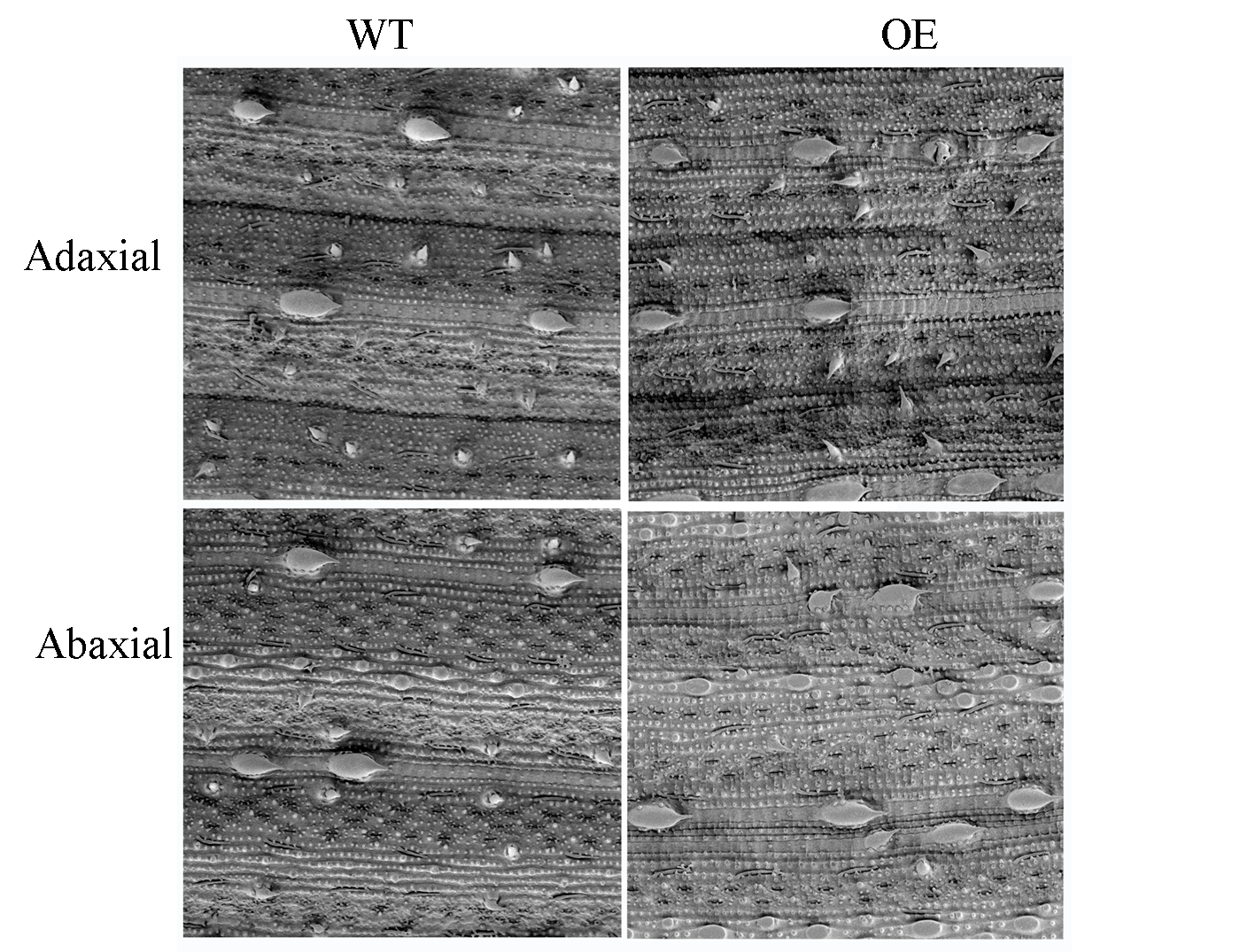


**Figure S4.**


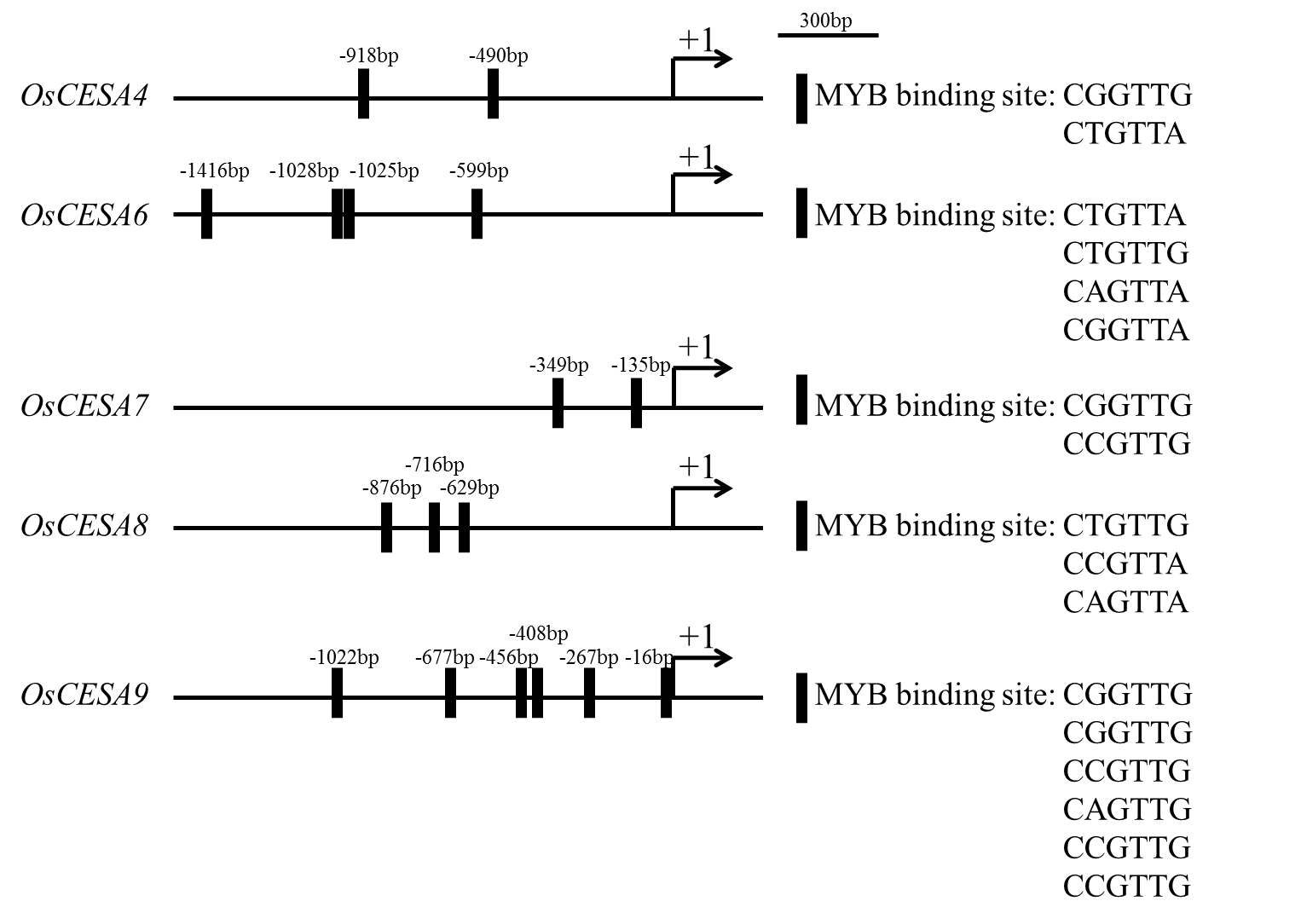


**Figure S5.**


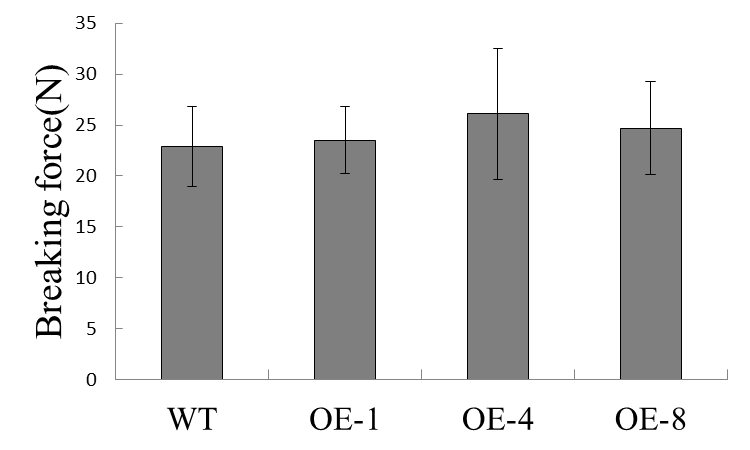


**Figure S6.**

**Table S2.** Expression levels of *CESA* genes in DGE analysis between wild type (WT) and *OsMYB103L* overexpressing plants (OE-1).

TPM, Transcript Per Million; FDR, False Discovery Rate.

| **Name** | **Locus** | **TPM** | | **Log2 Ratio** | **P-Value** | **FDR** | **Change** |
| --- | --- | --- | --- | --- | --- | --- | --- |
|  |  | **WT** | **OE** |  |  |  |  |
| OsMYB103L | LOC_Os08g05520 | 0.83 | 497.14 | 9.22 | 8.33E-05 | 3.80E-04 | Up |
| OsCESA1 | LOC_Os05g08370 | 9.69 | 21.46 | 1.15 | 7.50E-05 | 3.50E-04 | Up |
| OsCESA2 | LOC_Os03g59340 | 6.37 | 10.28 | 0.69 | 0.075 | 0.143 |  |
| OsCESA3 | LOC_Os07g24190 | 116.61 | 209.43 | 0.84 | 1.34E-13 | 2.27E-12 |  |
| OsCESA4 | LOC_Os01g54620 | 6.92 | 83.71 | 3.6 | 3.70E-10 | 3.92E-09 | Up |
| OsCESA5 | LOC_Os03g62090 | 3.05 | 0.6 | -2.35 | 0.02 | 0.04 | Down |
| OsCESA6 | LOC_Os07g14850 | 11.36 | 3.02 | -1.91 | 3.57E-05 | 1.80E-04 | Down |
| OsCESA7 | LOC_Os10g32980 | 4.99 | 16.32 | 1.71 | 3.06E-06 | 1.90E-05 | Up |
| OsCESA8 | LOC_Os07g10770 | 74.23 | 463.29 | 2.64 | 0 | 0 | Up |
| OsCESA9 | LOC_Os09g25490 | 0.01 | 0.6 | 5.91 | 0.22 | 0.32 | Up |
| OsCESA11 | LOC_Os06g39970 | 0.55 | 1.21 | 1.14 | 0.38 | 0.49 | Up |

**Table S3.** Primers of qRT-PCR used in this article.

| Name Primer sequence (5'-3') |
| --- |
| *OsMYB103L*_F CTTAGAAGATGGCCAAACAGCC  *OsMYB103L*_R TGGCCTCCAAGTTGGATGAT  *OsCESA1*_F CACTTATCGCCTATTGTGTGC  *OsCESA1*_R AAGAGATGGGCAGATGTGC  *OsCESA2*_F GGTATCCTTGAGATGAGGTGG  *OsCESA2*_R GCCTTTGAGGTGACAGTGAA  *OsCESA3*_F AAGTTCTTCGGTGGGCTCT  *OsCESA3*_R TTTCCAGGATGCCAGTAGC  *OsCESA4*_F TCTACGGTTCAGTCACAGAGG  *OsCESA4*_R AGCCATTTCAGACGACCA  *OsCESA5*_F GGACATCCATTCCACTATTGG  *OsCESA5*_R GCCTACACCACTCCATCTCAT  *OsCESA6*_F TTCTTCACATCTCTTCGCTG  *OsCESA6*_R CCTTCCAACCAAACCCTT  *OsCESA7*_F CTCCGTCGAGATCTTCATGAGC  *OsCESA7*_R CGCCAAATTGTTAAGCGTGG  *OsCESA8*_F TCCTTGAGATGAGGTGGAGT  *OsCESA8*_R TGGGATGAGAAGCGTTGT  *OsCESA9*_F CCTCTACGGCTACAAGAACG  *OsCESA9*_R GAAGAACAAACTCGCAAACG  *OsCESA11*_F GAGAGAGGCGATGTGCTTT  *OsCESA11*_R TCCTGTTCGTCGGAGTCAT  *ACTIN1*_F AGCAACTGGGATGATATGGA  *ACTIN1*_R CAGGGCGATGTAGGAAAGC |

1. Fang L, Zhao F, Cong Y, Sang X, Du Q, Wang D, Li Y, Ling Y, Yang Z, He G: **Rolling-leaf14 is a 2OG-Fe (II) oxygenase family protein that modulates rice leaf rolling by affecting secondary cell wall formation in leaves**. *Plant Biotechnol J* 2012, **10**(5):524-532.
